# *Interferon regulatory factor 5* genetic variants are associated with cardiovascular disease in patients with rheumatoid arthritis

**DOI:** 10.1186/ar4608

**Published:** 2014-07-10

**Authors:** Mercedes García-Bermúdez, Raquel López-Mejías, Fernanda Genre, Santos Castañeda, Javier Llorca, Carlos González-Juanatey, Alfonso Corrales, Begoña Ubilla, José A Miranda-Filloy, Trinitario Pina, Carmen Gómez-Vaquero, Luis Rodríguez-Rodríguez, Benjamín Fernández-Gutiérrez, Alejandro Balsa, Dora Pascual-Salcedo, Francisco J López-Longo, Patricia Carreira, Ricardo Blanco, Javier Martín, Miguel A González-Gay

**Affiliations:** 1 Institute of Parasitology and Biomedicine López-Neyra, IPBLN-CSIC, Parque Tecnológico de Ciencias de la Salud, Avenida del Conocimiento s/n, Armilla, 18100 Granada, Spain; 2 Epidemiology, Genetics and Atherosclerosis Research Group on Systemic Inflammatory Diseases, Rheumatology Division, IDIVAL, Avenida Cardenal Herrera Oria s/n, 39011 Santander, Spain; 3 Department of Rheumatology, Hospital Universitario la Princesa, IIS-Princesa, Diego de León 62, 28006 Madrid, Spain; 4 Department of Epidemiology and Computational Biology, School of Medicine, University of Cantabria, and CIBER Epidemiología y Salud Pública (CIBERESP), IDIVAL, Avenida Cardenal Herrera Oria s/n, 39011 Santander, Spain; 5 Cardiology Division, Hospital Universitario Lucus Augusti, Doctor Ochoa s/n, 27004 Lugo, Spain; 6 Department of Rheumatology, Hospital Universitario Lucus Augusti, Doctor Ochoa s/n, 27004 Lugo, Spain; 7 Department of Rheumatology, Hospital Universitario Bellvitge, Feixa Llarga s/n, 08907 Barcelona, Spain; 8 Department of Rheumatology, Hospital Clínico San Carlos, Profesor Martín Lagos s/n, 28040 Madrid, Spain; 9 Department of Rheumatology, Hospital Universitario La Paz, Paseo de la Castellana 261, 28046 Madrid, Spain; 10 Department of Rheumatology, Hospital General Universitario Gregorio Marañón, Doctor Esquerdo 46, 28007 Madrid, Spain; 11 Department of Rheumatology, Hospital Universitario 12 de Octubre, Avenida de Córdoba s/n, 28041 Madrid, Spain; 12 Cardiovascular Pathophysiology and Genomics Research Unit, School of Physiology, Faculty of Health Sciences, University of the Witwatersrand, 1 Jan Smuts Avenue Braamfontei, 2000 Johannesburg, South Africa

## Abstract

**Introduction:**

Rheumatoid arthritis (RA) is a complex polygenic inflammatory disease associated with accelerated atherosclerosis and increased cardiovascular (CV) disease risk. Interferon regulatory factor 5 (IRF5) is a regulator of type I interferon induction. Recently, researchers have described an association between multiple single-nucleotide polymorphisms of the *IRF5* gene and some rheumatic disorders. In this study, we aimed to evaluate whether three different haplotype blocks within the *IRF5* locus which have been shown to alter the protein function are involved in the risk of CV events occurring in Spanish RA patients.

**Methods:**

Three *IRF5* polymorphisms (rs2004640, rs2070197 and rs10954213) representative of each haplotype group were genotyped by performing TaqMan assays using a 7900HT Fast Real-Time PCR System with tissue from a total of 2,137 Spanish patients diagnosed with RA. Among them, 390 (18.2%) had experienced CV events. The relationship of *IRF5* genotypes and haplotypes to CV events was tested using Cox regression.

**Results:**

Male sex, age at RA diagnosis and most traditional risk factors (hypertension, dyslipidemia and smoking habit) were associated with increased risk for CV events in the RA population. Interestingly, a protective effect of both *IRF5* rs2004640 GG and *IRF5* rs10954213 GG genotypes against the risk for CV events after adjusting the results for sex, age at RA diagnosis and traditional CV disease risk factors was observed (hazard ratio (HR) = 0.6, 95% confidence interval (CI) = 0.38 to 0.92, *P* = 0.02; and HR = 0.58, 95% CI = 0.36 to 0.95, *P* = 0.03, respectively). Moreover, we detected a protective effect of the GTG haplotype against the risk for CV events after adjusting the results for potential confounding factors (HR = 0.72, 95% CI = 0.56 to 0.93, *P* = 0.012).

**Conclusions:**

Our results reveal that *IRF5* gene variants are associated with risk of CV events in patients with RA.

## Introduction

Rheumatoid arthritis (RA) is a chronic inflammatory rheumatic disease associated with an increased risk for cardiovascular (CV) events and CV disease–related deaths compared to the general population [1]. Because of that, adequate stratification of CV disease risk has special relevance in RA patients. Researchers in several studies have demonstrated that RA is an independent risk factor for premature heart disease [2]. This process is the result of a combined effect of traditional CV disease risk factors [3], the magnitude and severity of a chronic inflammatory response [4] and genetic factors located inside [4] and outside the human leukocyte antigen (HLA) region [5-8].

Type I interferons (IFNs) are signaling molecules involved in both innate and adaptive immunity. Misregulated expression of type I *IFN* genes has been observed in peripheral white blood cells of patients with several autoimmune diseases [9]. In this regard, interferon regulatory factor 5 (IRF5) has been involved in the regulation of type I *IFN* gene transcription [10,11]. This protein is critical for the production of proinflammatory cytokines [12], such as TNF-α, interleukin 12 (IL-12) and IL-6, following Toll-like receptor signaling. IRF5 also acts as a molecular switch that controls inflammatory mechanisms mediated by macrophage cells [12].

IRF5 protein is encoded by the *IRF5* gene, which is located in chromosome 7q32.1. Several genetic studies have described the relevance of multiple single-nucleotide polymorphisms (SNPs) of the *IRF5* gene in different rheumatic disorders, such as RA and lupus erythematosus [13,14]. In this context, three groups of correlated *IRF5* variants, designated as groups 1, 2 and 3, have been found to be independently associated with these inflammatory disorders and with different functional roles. Group 1 includes SNPs tagging a 30-bp in-frame insertion/deletion variant of exon 6 that alters protein stability. The association of group 2 SNPs seems to be explained by the T allele of *IRF5* rs2004640 polymorphism that allows the expression of an alternative isoform and is associated with significantly higher levels of *IRF5* expression. The association of group 3 SNPs is probably due to the rs10954213 A allele that creates an early polyadenylation site which leads to higher *IRF5* expression [14].

Taking into account all of these considerations together in the present study, we analyzed the potential role of one tagging SNP of each group of independent signals (rs2004640, rs2070197 and rs10954213) in the risk for CV disease in a large and well-characterized cohort of patients with RA.

## Methods

### Patients and study protocol

A set of 2,137 Spanish patients with RA were included in the present study. Blood samples were obtained from patients recruited from University Hospital Lucus Augusti (Lugo), Marqués de Valdecilla University Hospital (Santander), Hospital de Bellvitge (Barcelona) Hospital Clínico San Carlos, La Paz University Hospital, Hospital de La Princesa, University Hospital Gregorio Marañón and 12 de Octubre University Hospital (Madrid). Informed written consent was obtained from all participants. The study was approved by the ethics committees of Galicia for University Hospital Lucus Augusti; of Cantabria for Marqués de Valdecilla University Hospital; of Cataluña for Hospital de Bellvitge; and of Madrid for Hospital Clínico San Carlos, La Paz University Hospital, Hospital de La Princesa, University Hospital Gregorio Marañón and 12 de Octubre University Hospital. All the patients fulfilled the RA classification criteria published by the American College of Rheumatology in 1987 and 2010 [15,16]. In all the cases, patients were assessed for the *IRF5* rs2004640, rs2070197 and rs10954213 polymorphisms.

Data on the main demographic data, clinical characteristics, CV disease risk factors and CV events of patients enrolled in the study are shown in Table 1. Three hundred ninety (18.2%) of the patients had experienced CV events. Definitions of CV events and traditional CV disease risk factors were established as previously described [4,17].

**Table 1:** **Demographic and clinical characteristics**^**a**^

**Clinical features**	**% ( *n */ *N *) or mean ± SD**
Patients	2,137
Main characteristics	
Age at disease onset (yr)	53 ± 14.8
Follow-up duration (yr)	12.5 ± 8.25
Females (%)	75.1
Rheumatoid factor–positive^b^	68.3 (1,413/2,070)
Anti-CCP antibody–positive	57.3 (1,024/1,785)
Shared epitope–positive	62.6 (734/1,173)
Erosions	54.4 (866/1,591)
Extraarticular manifestations^c^	31.3 (481/1,537)
Cardiovascular risk factors	
Hypertension	40.3 (846/2,100)
Diabetes mellitus	13.1 (275/2,100)
Dyslipidemia	38.3 (805/2,100)
Obesity	19.6 (413/2,100)
Smoking habit	27.4 (576/2,100)
Patients with cardiovascular events (total)	18.2 (390/2,137)
Ischemic heart disease	8.5 (181/2,137)
Heart failure	5.7 (122/2,137)
Cerebrovascular accident	5.6 (120/2,137)
Peripheral arteriopathy	2.8 (60/2,137)

^a^CCP: Cyclic citrullinated peptide; SD: Standard deviation. ^b^At least two determinations were required for analysis of this result. ^c^Extraarticular manifestations of disease (if RA patients had at least one of the following manifestations: nodular disease: Felty’s syndrome, pulmonary fibrosis, rheumatoid vasculitis, secondary Sjögren’s syndrome) [4].

### Genotyping

Patient DNA was obtained from peripheral blood using standard methods. The *IRF5* rs2004640, rs2070197 and rs10954213 polymorphisms were genotyped with predesigned TaqMan SNP genotyping assays in a 7900HT Fast Real-Time PCR System according to the conditions recommended by the manufacturer (Applied Biosystems, Foster City, CA, USA). Negative controls and duplicate samples were included to check the accuracy of genotyping.

### Statistical analysis

The genotype data were checked for deviation from Hardy-Weinberg equilibrium (HWE) using a previously described method [18]. The relationship of genotypes, alleles and haplotypes to CV events that occurred in the follow-up was tested using Cox regression adjusted for sex, age at RA diagnosis and traditional CV disease risk factors. For that purpose, we used the most frequent genotype, allele and haplotype as reference variables. The end of follow-up was the first date of occurrence of the following: end of the study period, date of death or date of CV event. Follow-up time was estimated as the difference between the RA diagnosis date and the end of follow-up. Patients without CV events during the follow-up time and those who died by any non–CV-event–related cause were censored. The results are expressed as hazard ratios (HRs) with 95% confidence intervals (CIs). In order to have a reference population, 10,000 replications were generated by assigning CV events in randomized fashion to the actual studied population. The proportion of the risk for CV disease due to age at disease diagnosis, smoking history, hypertension and genetic variants at the *IRF5* locus among the RA patients was estimated as the Nelson-Aalen cumulative HR. Comparative values between the different genetic models of inheritance for *IRF5* polymorphisms were estimated using the Akaike Information Criterion (AIC). Statistical significance was defined as *P* ≤ 0.05, and all analyses were performed using Stata SE/12 statistical software (Stata Corp, College Station, TX, USA).

## Results

The *IRF5* rs2004640, rs2070197 and rs10954213 polymorphism genotype distributions were in Hardy-Weinberg equilibrium. The genotyping success rate was greater than 98% in all cases. Genotype and allele frequencies of the *IRF5* rs2004640, rs2070197 and rs10954213 polymorphisms were in agreement with the data in the HapMap database [19].

Table 2 describes those factors that were associated with the risk of CV disease in our cohort of RA patients. As expected, sex and most of the traditional CV disease risk factors (hypertension, dyslipidemia and smoking habit) were associated with increased risk of CV events (*P* < 0.05 in all cases) (Table 2). Interestingly, when we analyzed the *IRF5* rs2004640, rs2070197 and rs10954213 polymorphisms separately, we observed a protective effect of the *IRF5* rs2004640 GG genotype against the risk of CV events after adjusting the results for sex, age at RA diagnosis and traditional CV disease risk factors (hypertension, diabetes mellitus, dyslipidemia, obesity and smoking habit) (HR = 0.6, 95% CI = 0.38 to 0.92, *P* = 0.02) (Table 2). Additionally, the risk of CV disease was decreased in the group of RA patients who carried the *IRF5* rs10954213 GG genotype after we adjusted the results for potential confounders (HR = 0.58, 95% CI = 0.36 to 0.95, *P* = 0.03) (Table 2). These results were also significant after we estimated by simulation (*P* = 0.034 and *P* = 0.049 for the *IRF5* rs2004640 GG and rs10954213 GG genotypes, respectively) (Table 2). However, we found no statistically significant differences when we assessed the *IRF5* rs2070197 polymorphism according to the presence or absence of CV events (Table 2).

**Table 2:** **Factors associated with cardiovascular events in a series of 2,137 rheumatoid patients**^**a**^

**Variables**		**HR (95% CI)**	***P*-values**	***P*-values**^**b**^
Males (reference: females)		1.42 (1.07 to 1.87)	0.015	
Age at RA diagnosis (by each year)		1.07 (1.06 to 1.09)	<0.001	
Hypertension		1.40 (1.029 to 1.92)	0.032	
Diabetes mellitus		1.18 (0.83 to 1.68)	0.344	
Obesity		0.93 (0.67 to 1.31)	0.702	
Dyslipidemia		1.42 (1.05 to 1.93)	0.022	
Smoking		1.58 (1.26 to 1.99)	<0.001	
rs2004640^c^	TT	1 (reference)	–	–
	TG	1.07 (0.79 to 1.45)	0.664	0.670
	GG	0.59 (0.38 to 0.92)	0.020	0.034
rs2070197^c^	TT	1 (reference)	–	–
	TC	1.16 (0.69 to 1.95)	0.58	–
	CC	1.32 (0.32 to 5.52)	0.39	–
rs10954213^c^	AA	1 (reference)	–	–
	AG	0.95 (0.71 to 1.26)	0.73	0.738
	GG	0.58 (0.36 to 0.95)	0.03	0.049

^a^CI: Confidence interval; HR: Hazard ratio; RA: Rheumatoid arthritis. ^b^*P*-values estimated on the basis of 10,000 replications. ^c^Adjusted for sex, age at time of rheumatoid arthritis diagnosis and traditional cardiovascular disease risk factors (hypertension, diabetes mellitus, dy*s*lipidemia*,* obesity, smoking habit).

In a further step, we combined the three polymorphisms to create haplotypes (Table 3). As shown in Table 3, we detected a protective effect of the GTG haplotype after adjusting the results for sex, age at RA diagnosis and traditional CV disease risk factors (HR = 0.72, 95% CI = 0.56 to 0.93, *P* = 0.012).

**Table 3:** **Results of haplotype analysis in rheumatoid arthritis patients with vs. without cardiovascular disease**^**a**^

**Haplotypes**			**HR (95% CI)**^**b**^	**P-value**^**b**^
**rs2004640**	**rs2070197**	**rs10954213**		
T	T	A	1 (reference)	–
G	T	G	0.72 (0.56 to 0.93)	0.012
G	T	A	0.85 (0.61 to 1.18)	0.34
T	C	A	1.14 (0.75 to 1.74)	0.52

^a^CI: confidence interval; HR: hazard ratios. ^b^Adjusted for sex, age at time of rheumatoid arthritis diagnosis and traditional cardiovascular disease risk factors (hypertension, diabetes mellitus, dyslipidemia*,* obesity and smoking habit).

Additional file 1: Table S1 describes the proportion of the CV disease risk disease for RA patients due to age at disease diagnosis, smoking history, hypertension and the genetic variants at the *IRF5* locus. Additional file 2: Table S2 displays the genetic model of inheritance of our analysis. Interestingly, as shown in Additional file 1: Table S1, the presence of GG genotypes in the *IRF5* rs2004640 and rs1095213 polymorphisms decreased CV disease risk in 0.7% and 1% of patients, respectively (Additional file 1: Table S1). In addition, smoking, age at disease diagnosis and presence of hypertension increased the risk of CV disease (Additional file 1: Table S1). Further, as shown in Additional file 2: Table S2, the AIC estimation for the recessive model showed the lower level in the *IRF5* rs2004640 and rs1095213 polymorphisms, which made it the preferable model for our study (Additional file 2: Table S2).

## Discussion

CV disease is the most common cause of premature mortality in patients with RA [1,2]. The augmented CV disease mortality observed in this pathology is the result of a compound effect mediated by traditional CV disease risk factors and chronic inflammation [3,4]. Because genes have also been associated with an increased risk of CV disease in RA, research in the past several years has been focused on the search for genetic markers that may improve the identification of RA patients at risk of experiencing CV events [4-8].

Outside the HLA region, IFN pathway genes, which encode cytokines with critical modulatory effects on innate and adaptive immunity, have been shown to represent a key component of the genetic network leading to autoimmune processes. In this context, several polymorphisms in the *IRF5* gene (a regulator of type I IFN induction) [10,11] are associated with an increased risk of immune-mediated diseases [13,14]. Because of that, in this study we analyzed three genetic variants (*IRF5* rs2004640, rs2070197 and rs10954213) as representatives of three different *IRF5* haplotype blocks [14]. To the best of our knowledge, our study constitutes the largest series of RA patients assessed for the potential influence of *IRF5* polymorphisms in the risk of CV disease. Interestingly, when we studied each of the polymorphisms separately, our results revealed a protective effect of the *IRF5* rs2004640 GG and *IRF5* rs10954213 GG genotype variants against the risk of CV events in RA. Moreover, when we analyzed all the genetic variants together to create haplotypes, our results revealed a protective effect of the GTG haplotype (the one that carries both G alleles of *IRF5* rs2004640 and rs10954213 polymorphisms) against the risk of CV disease. Because the protective effect of the mutant allele of the *IRF5* rs2004640 and rs1095213 polymorphisms is seen only in homozygosis, we can conclude that our study is adjusted to a recessive model of inheritance. Additionally, the results derived from our study are in accordance with those obtained in other pathologies [20,21]. In this context, the results of a recent study of individuals diagnosed with RA revealed an association between the *IRF5* rs2004640 polymorphism and subclinical atherosclerosis [22].

The results obtained in this study provide additional evidence on the potential role that genetic factors may play in the development of CV disease in RA. The search for genetic markers associated with CV disease in RA may be important to establishing a better characterization of RA patients at risk of CV disease. Improved understanding of these underlying genetic may be useful to establish future therapeutic targets to decrease the risk of CV disease in RA patients.

## Conclusion

Our results reveal that *IRF5* gene variants are associated with CV disease in RA patients.

## Abbreviations

ACR: American College of Rheumatology; Anti-CCP: Anti–cyclic citrullinated protein/peptide antibody; CI: Confidence interval; CV: Cardiovascular; HLA: Human leukocyte antigen; HR: Hazard ratio; IFN: Interferon; IL: Interleukin; IRF: Interferon regulatory factor; RA: Rheumatoid arthritis; SD: Standard deviation; SLE: Systemic lupus erythematosus; SNP: Single-nucleotide polymorphism; TNF: Tumor necrosis factor.

## Competing interests

The authors declare that they have no competing interests.

## Authors’ contributions

MGB, RLM and FG carried out genotyping, participated in the study design and data analysis and helped to draft the manuscript. SC and BFG were involved in the acquisition and interpretation of data and in revising the manuscript critically for important intellectual content. JL carried out data analysis and interpretation. AC and CGJ performed the carotid ultrasound examination and were involved in the acquisition and interpretation of data as well as study coordination, and they helped to draft the manuscript. BU, JAMF, TP, CGV, LRR, AB, DPS FJLL, PC and RB participated in the acquisition and interpretation of data and helped to draft the manuscript. JM and MAGG made substantial contributions to the study’s conception and design, data acquisition and study coordination, and they helped to draft the manuscript. All authors read and approved the final manuscript.

## Authors’ information

JM and MAGG share senior authorship of this article.

## Supplementary Material

Additional file 1: Table S1 Proportion of the CV risk for RA patients due to age, smoking history, hypertension and the genetic variants at the *IRF5* locus at 5 years. Click here for file

Additional file 2: Table S2 Comparison between different genetic models of inheritance for *IRF5* polymorphisms. Click here for file
