# Supplementary material for: Interferon regulatory factor 5 genetic variants are associated with cardiovascular disease in patients with rheumatoid arthritis
Source: Arthritis Res Ther. 2014 Jul 10;16(4):R146. doi: 10.1186/ar4608 (PMC4227041; doi:10.1186/ar4608)
Supplement: Additional file 1: Table S1 — Proportion of the CV risk for RA patients due to age, smoking history, hypertension and the genetic variants at the IRF5 locus at 5 years. [file ar4608-S1.doc]

**Supplementary table 1: Proportion of the CV risk for RA patients due to age, smoking history, hypertension and the genetic variants at the *IRF5* locus at 5 years.**

| **Variable** | **Category** | **5-year cumulative risk (%)** |
| --- | --- | --- |
| Age at RA diagnosis | First quartile | 0.2 |
|  | Second quartile | 2.1 |
|  | Third quartile | 5.5 |
|  | Forth quartile | 9.8 |
| Smoking | Never | 3.5 |
|  | Former | 4.9 |
|  | Current | 5.5 |
| Arterial hypertension | No | 2.8 |
|  | Yes | 6.3 |
| rs2004640 | TT | 4.3 |
|  | TG | 4.3 |
|  | GG | 3.6 |
| rs1095213 | AA | 4.7 |
|  | AG | 4.1 |
|  | GG | 3.7 |

RA: rheumatoid arthritis; CV: cardiovascular
