# Supplementary material for: Interferon regulatory factor 5 genetic variants are associated with cardiovascular disease in patients with rheumatoid arthritis
Source: Arthritis Res Ther. 2014 Jul 10;16(4):R146. doi: 10.1186/ar4608 (PMC4227041; doi:10.1186/ar4608)
Supplement: Additional file 2: Table S2 — Comparison between different genetic models of inheritance for IRF5 polymorphisms. [file ar4608-S2.doc]

**Supplementary table 2**: Comparison between different genetic models of inheritance for *IRF5* polymorphisms.

|  | **rs2004640** | | **rs1095213** | |
| --- | --- | --- | --- | --- |
| **Model** | **Genotypes compared** | **AIC** | **Genotype reference** | **AIC** |
| Co-dominant | TT (ref)/TG/GG | 2478.6 | AA (ref)/AG/GG | 2492.6 |
| Dominant | TT (ref)/TG or GG | 2485.5 | AA (ref)/AG or GG | 2494.7 |
| Recessive | TT or TG (ref)/GG | 2476.8 | AA or AG (ref)/GG | 2490.7 |
| Over-dominant | TT or GG (ref)/TG | 2482.3 | AA or GG (ref)/AG | 2495.9 |
| Additive | TT/TG/GG as continuous variable | 2481.7 | AA/AG/GG as continuous variable | 2492.4 |

AIC: Akaike Information Criterion.
